# Electronic Brainstorming With a Chatbot Partner: A Good Idea Due to Increased Productivity and Idea Diversity

**DOI:** 10.3389/frai.2022.880673

**Published:** 2022-09-23

**Authors:** Britt Wieland, Jan de Wit, Alwin de Rooij

**Affiliations:** ^1^Department of Communication and Cognition, Tilburg Center for Cognition and Communication, Tilburg School of Humanities and Digital Sciences, Tilburg University, Tilburg, Netherlands; ^2^St. Joost School of Art and Design, Avans University of Applied Sciences, Breda, Netherlands

**Keywords:** chatbot, brainstorming, creativity support tools, evaluation apprehension, human-machine teaming

## Abstract

Brainstorming is a creative technique that fosters collaboration to enhance idea generation. The occurrence of evaluation apprehension, a fear of being evaluated negatively by others, however, can stymy brainstorming. How the advantages of collaboration can be leveraged while evaluation apprehension is prevented is an open scientific and practical problem. In this brief research report, it is proposed that chatbots could provide a solution. Chatbots can be designed to share ideas with their users, facilitating inspiration. Compared to human beings, chatbots are also perceived as possessing limited agency and evaluative capacity. This could reduce evaluation apprehension. Given that chatbots are often embedded in a textual chat interface, social cues (picture, name, and description) can reinforce the perceived chatbot identity, enhancing its alleged effects on evaluation apprehension and subsequently on brainstorming performance. These conjectures were tested in an online 2 × 2 between-subjects experiment (*n* = 120) where people were instructed to brainstorm with a partner that was framed as either a chatbot or human being (but followed the same automated script), with or without the presence of social cues. The results showed that brainstorming with a chatbot led participants to produce more ideas, with more diversity than brainstorming with an alleged human being. Social cues enhanced the effect on idea diversity, but only with the chatbot. No significant effects on evaluation apprehension were found. The contribution of this study is therefore that chatbots can be used for effective human–machine teaming during brainstorming, but this enhancement is not explained by its effects on evaluation apprehension.

## Introduction

Brainstorming is an often-used creative technique that supports creativity and productivity during idea generation by fostering collaboration (Osborn, 1957). The assumption is that generating many, highly diverse ideas, while explicitly not imposing any constraints upon oneself or others, will ultimately lead to quality. This is because ideas generated by one person, for example, can contain information and activate semantic categories that are not otherwise accessible to another (Nijstad and Stroebe, 2006), enabling people to inspire more, and more diverse ideas in one another (Osborn, 1957; Diehl and Stroebe, 1987). However, one critical disadvantage of collaboration is evaluation apprehension: the fear of being judged negatively by others, which causes people to share fewer ideas during a brainstorm (Diehl and Stroebe, 1987; Bordia et al., 2006). Given the widespread reliance on brainstorming during the creative and innovation process of many businesses and organizations, it is worthwhile to explore how to use digital tools that can enable the benefits of collaboration, while also reducing evaluation apprehension (Bittner et al., 2019; Tavanapour et al., 2019; Geerts et al., 2021; Gozzo and de Rooij, 2021). In this brief research report, it is proposed that chatbots, an emerging technology designed to interact with users through natural language (Hendriks et al., 2020), could be designed as alternative brainstorming partners in a manner that addresses this open scientific and practical problem.

Evaluation apprehension can arise from fearing the negative social consequences of certain actions (e.g., disapproval), fearing giving a bad impression and from protecting one's self-image (Bordia et al., 2006). Therefore, people may not share (all) their ideas with the group to prevent these (imagined) negative consequences (Diehl and Stroebe, 1987; Zhou et al., 2019). Zhou et al. (2019), for example, found that evaluation apprehension especially limits people to share their most original ideas. Therefore, evaluation apprehension can negatively affect the number and diversity of the ideas generated during brainstorming (Diehl and Stroebe, 1987; Zhou et al., 2019). Collaborating with a chatbot could reduce evaluation apprehension because these technologies, when compared to human beings, have limited agency (cf. Geerts et al., 2021; Gozzo and de Rooij, 2021). A chatbot is typically not thought of as judgmental and it is unlikely to have an ability to incur any negative social consequences upon its users (Oh et al., 2018; Lee et al., 2020). At the same time, chatbots could provide a social facilitation effect because they can interactively brainstorm with the user in real-time (Paulus et al., 2013), although it is yet unclear to what extent this also evokes social comparison, where an upward comparison could potentially lead to more and relatively diverse ideas (Dugosh and Paulus, 2005; Michinov et al., 2015).

Emerging empirical evidence supports our conjecture. Gozzo and de Rooij (2021) found that when a scripted collaboration on an association task was framed as a collaboration with a general-purpose AI, compared to with another person, evaluation apprehension was reduced. Furthermore, Hwang and Won (2021) found that people generated more ideas during an idea generation task when they engaged in a scripted collaboration with a chatbot. The participants in the chatbot condition suggested that they could express their ideas more freely compared to the participants who believed they collaborated with a human being (for which interactions were also scripted), possibly suggesting reduced evaluation apprehension.

Brainstorming with a human or chatbot brainstorming partner can be done *via* an online chat interface (e.g., Gozzo and de Rooij, 2021). According to Hendriks et al. (2020), the identity that is presented online is leading for the perception that people have of the identity of their interaction partner. Before an online chat interaction, the identity of an interaction partner can be framed through a brief introduction. During an online chat interaction, the identity of the interaction partner can be reinforced through social cues (Araujo, 2018; Go and Sundar, 2019). Social cues are verbal or non-verbal cues from an interaction partner that help in forming an impression of this interaction partner (e.g., name, profile picture, and short description; Go and Sundar, 2019). Go and Sundar (2019) found that the use of social cues for chatbots can reinforce their robotic nature and reduce the perceived human agency. In the context of collaborative brainstorming with a chatbot, social cues could therefore help reduce evaluation apprehension. Taken together, these conjectures suggest that chatbots can be designed to support brainstorming by keeping the advantages of collaboration, i.e., inspiration by sharing (pre-defined) ideas, while reducing one critical disadvantage, i.e., evaluation apprehension by having limited perceived human agency (Bittner et al., 2019; Gozzo and de Rooij, 2021). Given that evaluation apprehension predicts a reduced number and diversity of ideas during brainstorming (Diehl and Stroebe, 1987; Zhou et al., 2019), it follows that a reduction of evaluation apprehension by brainstorming with a chatbot will cause higher productivity and idea diversity. In addition, the availability of social cues may moderate this alleged effect because it reinforces the perceived identity of the brainstorming partner. This leads us to propose the following working hypothesis:

*Brainstorming with a partner framed as a chatbot, compared to as a human being, causes the generation of more ideas and more diverse ideas, which effect is mediated by a decrease in evaluation apprehension and moderated by the availability of social cues*.

## Methods

To test the working hypothesis an online experiment was conducted with a between-subjects 2 × 2 factorial design, where participants were asked to brainstorm with a partner that was framed as chatbot or human being in a chat interface, with or without social cues.

### Participants

One hundred twenty people participated in the experiment (*M*_*age*_ = 22.02, *SD*_*age*_ = 2.65, 82 females, 38 males). Participants were recruited from the human subject pool of Tilburg University and the researchers' network. Participants recruited from the human subject pool received course credits. All participants were Dutch native speakers. They self-reported moderate to high creative ability (*M* = 3.70, *SD* = 0.56) as measured with the Short Scale of Creative Self (Karwowski, 2016). Creative ability did not differ significantly between experimental conditions, *F*_(3,116)_ = 0.327, *p* = 0.81.

### Materials and Measures

The materials and measures are available as Supplementary material.

#### Brainstorming Task

The participants were asked to perform a brainstorming task following Osborn's (1957) brainstorming rules, and in collaboration with an assigned brainstorming partner. The participants were asked to generate ideas about the problem statement: “How can we deal with the threat of overpopulation in the Netherlands and the rest of the world?” This topic was chosen because of its topicality and because it could easily lead to ideas that may be taboo (Campbell, 2012). This was assumed to enable the elicitation of evaluation apprehension (Diehl and Stroebe, 1987). Participants shared their ideas one by one in a chat interface with the brainstorming partner. The layout of the chat window was based on the layout of chat windows of existing chat services, such as Facebook Messenger, and it was embedded into the Qualtrics questionnaire program.

#### Manipulations: Brainstorming Partner Identity and Social Cue Availability

**Figure 1:**
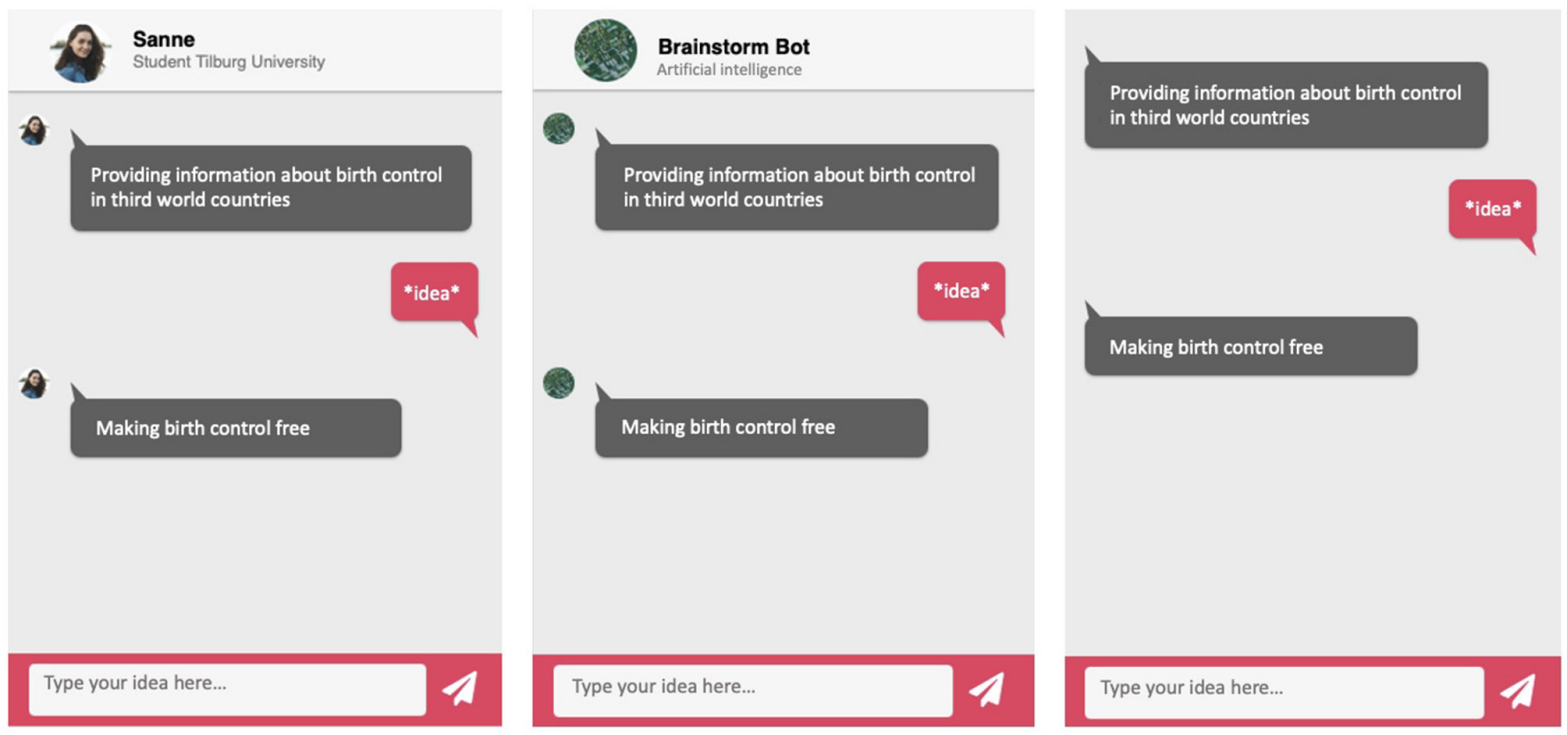
The manipulations of the chat interface.

Before the start of the brainstorm task, a brainstorming partner was introduced (framed) as either a chatbot or a human being by random assignment (see Table 1). The introduction contained a name and description of the identity of the brainstorming partner. The introductions used were based on the introductions of the interaction partners in Araujo (2018) and Hendriks et al. (2020). The availability of social cues in the chat interface was also randomized. In the condition with social cues, the chatbot's identity was emphasized by a profile picture, name and a short description. These social cues are often used in chat windows that are used in practice, such as the popular collaborative platform Slack (Rietz et al., 2019). The human chatbot was presented as a male or female fellow student performing university tasks (i.e., a student assistant). See Figure 1 for further details on the manipulations and chat interface.

**Table 1:** The introduction (framing) of the brainstorming partners.

**Chatbot brainstorming partner**	**Human brainstorming partner**
“You will brainstorm together with artificial intelligence in the form of a chatbot for 10 min, *via* a chat window about the problem statement: How can we deal with the threat of overpopulation in the Netherlands and the rest of the world? A chatbot is a technology that is based on prescribed rules and it uses natural language. The chatbot will share ideas with you that are based on previously collected data. Your ideas are visible to the chatbot. You can share your ideas by clicking the send icon in the chat window. Send 1 idea per message. You don't have to wait for an idea from the chatbot before sending a new idea. The chat window will close automatically after 10 min.”	“You will brainstorm together with a student assistant from Tilburg University for 10 min, *via* a chat window about the problem statement: How can we deal with the threat of overpopulation in the Netherlands and the rest of the world? You can share your ideas by clicking the send icon in the chat window. Send 1 idea per message. You and the other student will receive each other's ideas. You don't have to wait for an idea from the other student before sending a new idea. The chat window will close automatically after 10 min.”

#### The Script

The brainstorming partners, while introduced as a human or chatbot, were the same automated script. The script sent timed messages to the participant, without interpreting or using their responses. This made a larger scale experiment possible. Out of 71 ideas generated by test participants (*n* = 5; *M*_*age*_ = 32.2, *SD*_*ag*e_ = 14.55, 4 females, 1 male) *via* WhatsApp prior to the experiment, 14 ideas were included in the script, consisting of the five most frequently mentioned ideas plus nine randomly selected unique ideas. The number, order, and timing of when the script sent ideas to the participants were based on the averages of the brainstorming sessions with test participants, which also lasted 10 min. This was done to support believability of the brainstorming partners. The scripted brainstorming partners automatically provided one-time feedback on the ideas of the subjects (“What kind of ideas are those…”). This was done to make the participants aware that their submitted ideas were visible to and could be evaluated by their brainstorming partner, which was assumed to enable evaluation apprehension. After providing feedback, the chatbot excused itself for breaking the rules.

#### Assessment of Evaluation Apprehension

Evaluation apprehension was measured using the seven-item five-point Likert scale (1 = strongly disagree, 5 = strongly agree) by Bolin and Neuman (2006). The items of the scale have been adapted to the design of the experiment. For example, the item “*I didn't send all my ideas, because I didn't want the group to think I was weird or crazy*” was changed to: “*I didn't send all my ideas, because I didn't want my brainstorming partner to think I was weird or crazy*.” Also, an irrelevant item (“Within the group we took everyone's ideas into account”) was replaced by: “I was afraid that my brainstorming partner would think badly of me.” The participants were asked to what extent they agreed or disagreed with statements about the brainstorming session. Cronbach's alpha showed a questionable internal consistency, α = 0.69. After removing the item “*My brainstorming partner and I looked at each other's ideas*,” the internal consistency was acceptable, α = 0.72.

#### Assessment of Brainstorming Performance

Productivity during the brainstorming task was measured by adding up the number of ideas generated by each participant (Diehl and Stroebe, 1987). Idea diversity was measured by adding up the number of different semantic categories found in the ideas generated by a single participant (inspired by Nijstad and Stroebe, 2006). For example, the idea “Legalize abortion and euthanasia” consists of three unique categories (i.e., abortion, euthanasia, legalize). Another idea by the same participant, “Making abortion accessible,” would then consist of one new, unique semantic category (i.e., making something accessible). Broadly speaking, the categories consisted of actions (e.g., reducing, educating, and stimulating), subjects (e.g., contraceptives and housing), and contextual factors (e.g., in developing countries, world-wide). Productivity and idea diversity were annotated for 30 participants (25%) by an additional researcher, resulting in Krippendorff's α = 0.99 (productivity) and 0.88 (diversity).

#### Assessment of Social Presence, Perceived Humanness, and Perceived Homophily

Perceived humanness, homophily, and social presence were included in the study as additional measures. It was expected that the reported perceived humanness and homophily would be higher for the human brainstorming partner than for the chatbot brainstorming partner (Go and Sundar, 2019; Hendriks et al., 2020). Moreover, it was expected that the reported social presence would be higher in the conditions with social cues compared to the conditions without social cues (Go and Sundar, 2019). The social presence scale was based on the scales used by Go and Sundar (2019) and Hendriks et al. (2020) in their chatbot studies, and consisted of five items rated on a five-point Likert scale (1 = strongly disagree, 5 = strongly agree). Cronbach's alpha showed good internal consistency, α = 0.84. Hendriks et al.'s (2020) six-item seven-point Likert scale of perceived humanness also showed an acceptable internal consistency, α = 0.75. As did Go and Sundar's (2019) four-item five-point Likert scales (1 = completely disagree, 5 = completely agree) used to assess perceived homophily, α = 0.87.

#### Demographics and Individual Differences

To capture relevant sample characteristics participants also reported their age and gender, and filled in the Short Scale of Creative Self (Karwowski, 2016; α = 0.87). The results are reported in the participants subsection.

### Procedure

The experiment was conducted online *via* Qualtrics. The participants read information about the experiment and provided informed consent prior to participating. Information about the actual purpose of the study was initially omitted. The participants were then asked to answer questions regarding their demographics and creativity. This was followed by the instruction of the brainstorming task in which the brainstorming rules of Osborn (1957) were explained. Then, either a chatbot or human being was introduced as the participant's brainstorming partner. Hereafter, the participants started the brainstorming task in the chat interface, where social cues were either available or not. The brainstorming task lasted 10 min. Then, the participants completed questionnaires related to evaluation apprehension, social presence, homophily, and perceived humanness. After completing the questionnaires, the participants were debriefed and thanked for their participation.

## Results

To provide insight into the general characteristics of the dataset the descriptive statistics were calculated. These are presented in Table 2. To test whether brainstorming with a partner framed as chatbot, compared to as a human being, causes the generation of more ideas and more diversity, and whether this effect is mediated by a decrease in evaluation apprehension and moderated by the availability of social cues, two moderated mediation analyses were calculated using Hayes' bootstrapping method (Hayes, 2017). Both analyses were calculated with brainstorming partner identity as the independent variable, social cue availability as the moderator of the effect of brainstorming partner on evaluation apprehension, and evaluation apprehension as the mediator. The models differed with productivity and idea diversity specified as the dependent variable. The results showed no significant effect of brainstorming partner on evaluation apprehension, *b* = −0.364, *se* = 0.438, *p* = 0.408, 95% CI [−1.232; 0.504], which effect was thus not significantly moderated by social cue availability, moderation index = −0.178, *se* = 0.333, 95% CI [−1.057; 0.284], and did not show an indirect effect of brainstorming partner on productivity *via* evaluation apprehension, *b* = −0.767, *se* = 0.595, 95% CI [−1.946; 0.411]. However, the results did show a significant direct effect of brainstorming partner on productivity, *b* = 4.199, *se* = 0.852, *p* < 0.001, 95% CI [2.512; 5.886]. That is, the participants who brainstormed with a partner framed as chatbot (*M* = 12.92, *SD* = 5.35) generated significantly more ideas on average than the subjects who brainstormed with a partner framed as human (*M* = 8.72, *SD* = 3.79). Similarly, the results showed no significant indirect effect of brainstorming partner on idea diversity *via* evaluation apprehension, *b* = −2.199, *se* = 1.328, 95% CI [−4.830; 0.431], and thus this relationship could also not be moderated by social cue availability, moderation index = −0.512, *se* = 0.857, 95% CI [−2.736; 0.721], but rather showed a significant direct effect of brainstorming partner on idea diversity, *b* = 7.735, se = 1.901, *p* < 0.001, 95% CI [3.969; 11.500]. That is, the participants who brainstormed with a partner framed as chatbot used significantly more different semantic categories in their ideas (*M* = 33.31, *SD* = 13.09) than the participants who brainstormed with a partner framed as human (*M* = 22.18, *SD* = 9.23). These findings lend partial support for the working hypothesis. Chatbots can be used to enhance brainstorming, but this enhancement is not explained by its effects on evaluation apprehension.

**Table 2:** Means and standard deviations (between parentheses).

**Variable**		**Chatbot brainstorming partner**	**Human brainstorming partner**	**Total**
1. Social presence	With social cues	2.57 (1.07)	2.39 (0.88)	2.48 (0.97)
	Without social cues	2.14 (0.73)	2.35 (0.71)	2.24 (0.72)
	Total	2.33 (0.91)	2.37 (0.79)	
2. Homophily	With social cues	2.33 (0.98)	2.21 (0.80)	2.27 (0.88)
	Without social cues	2.14 (0.73)	2.24 (0.89)	2.19 (0.80)
	Total	2.22 (0.84)	2.23 (0.84)	
3. Perceived humanness	With social cues	4.12 (0.93)	4.01 (1.03)	4.06 (0.97)
	Without social cues	3.84 (0.75)	4.01 (1.03)	3.92 (0.89)
	Total	3.96 (0.93)	4.01 (1.02)	
4. Evaluation apprehension	With social cues	2.47 (0.78)	2.57 (0.71)	
	Without social cues	2.65 (0.77)	2.58 (0.67)	
	Total	2.57 (0.77)	2.58 (0.68)	
5. Productivity	With social cues	14.31 (5.99)	8.41 (3.81)	
	Without social cues	11.85 (4.62)	9.00 (3.80)	
	Total	12.92 (5.35)	8.72 (3.79)	
6. Idea diversity	With social cues	33.31 (13.09)	21.41 (8.86)	
	Without social cues	27.17 (9.44)	22.90 (9.64)	
	Total	29.83 (11.48)	22.18 (9.23)	

**Figure 2:**
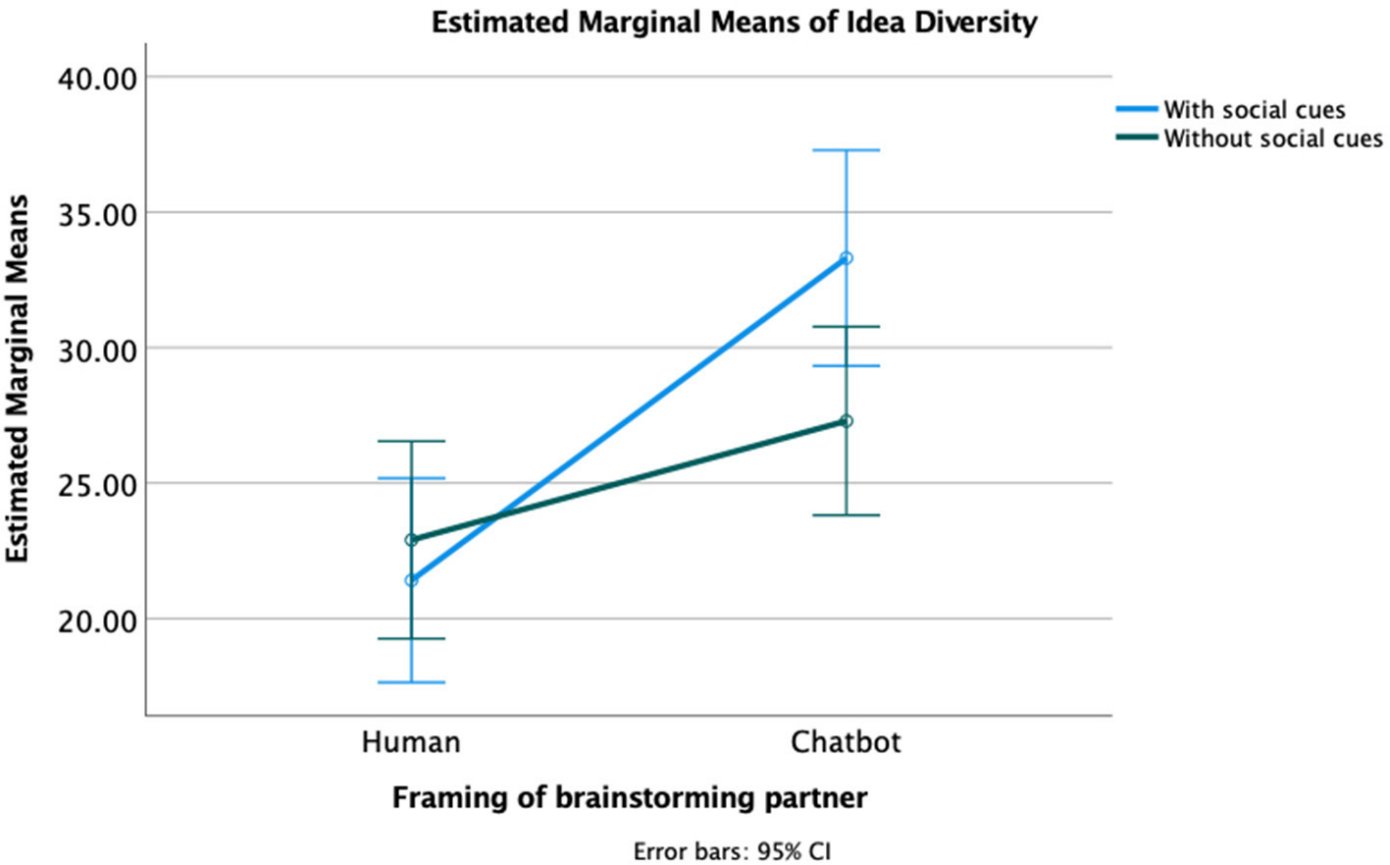
The interaction effect between brainstorming partner and social cues on idea diversity.

To further explore the significant direct effects of brainstorming partner on productivity and idea diversity, two factorial ANOVAs were calculated, both with brainstorming partner identity and social cue availability as the independent variables, and one with productivity and one with idea diversity as the dependent variable. The results showed a significant interaction effect between brainstorming partner and social cues on idea diversity, *F*_(1,116)_ = 3.99, *p* = 0.048, visualized in Figure 2. The participants with a brainstorming partner framed as chatbot used significantly more semantic categories in their ideas in the social cues condition (*M* = 33.31, *SD* = 13.09) than in the condition without social cues (*M* = 27.17, *SD* = 9.44). For the participants with a brainstorming partner framed as human, this was the other way around: the participants in condition without social cues (*M* = 22.90, *SD* = 9.64) used more semantic categories than the subjects in condition with social cues (*M* = 21.41, *SD* = 8.86). A similar pattern was found for productivity, but this interaction effect was not significant, *F*_(1,116)_ = 3.26, *p* = 0.073. These findings suggest that social cues can be used to enhance the effectiveness of brainstorming with a chatbot.

To explore the characteristics of the manipulations, factorial ANOVAs were calculated with brainstorming partner identity and social cue availability as the independent variables, and individually with perceived social presence, humanness and homophily as the dependent variables. The results showed no significant difference in perceived social presence when social cues were present (*M* = 2.48, *SD* = 0.97) or not (*M* = 2.34, *SD* = 0.72), *F*_(1,116)_ = 2.34, *p* = 0.129. Furthermore, no significant difference was found for homophily between brainstorming with the chatbot (*M* = 2.22, *SD* = 0.84) or human being (*M* = 2.23, *SD* = 0.84), *F*_(1,116)_ = 0.01, *p* = 0.938. Similarly, there was no significant difference for perceived humanness when brainstorming with the alleged human brainstorming partner (*M* = 4.01, *SD* = 1.02) compared to the chatbot brainstorming partner (*M* = 3.96, *SD* = 0.93), *F*_(1,116)_ = 0.03, *p* = 0.853. Thus, the perceived social presence, humanness and homophily of the brainstorming partner did not differ significantly between the conditions.

## Discussion

The presented study was conducted to explore whether brainstorming with a chatbot, compared to with a human being, causes the generation of more and relatively diverse ideas, and whether this effect is mediated by a decrease in evaluation apprehension and moderated by the availability of social cues.

The results lend partial support for the working hypothesis. Brainstorming with a chatbot, compared to with an alleged human being, caused participants to generate more ideas, with more diversity. However, this effect was not mediated by evaluation apprehension, nor was the relationship between brainstorming partner and evaluation apprehension moderated by social cue availability. These findings echo those by Geerts et al. (2021), but contrast to some extent with related research by Gozzo and de Rooij (2021), where collaboratively generating associations with a general-purpose AI reduced evaluation apprehension; and with Hwang and Won's (2021) finding that generating ideas together with a chatbot led people to suggest that they could express their ideas more freely compared to the participants who thought they collaborated with a human being. One explanation for the latter difference is that free expression may not be the result of reduced evaluation apprehension, but rather of increased behavioral disinhibition. Another explanation might relate to the perception of the chatbot and human identity in the present study. No differences in perceived humanness and homophily between the chatbot and human conditions were found, which may have resulted in social comparisons occurring in both conditions (Dugosh and Paulus, 2005; Michinov et al., 2015). Speculatively, this may be caused by the human-like conversational style, messaging app-like design of the interface, or the fact that the ideas shared in the chatbot condition require real-world knowledge that we typically associate with human–human interaction (cf. Araujo, 2018; Hendriks et al., 2020; Lee et al., 2020).

The finding that people generate more and diverse ideas when they believe they are brainstorming with a chatbot, rather than with a human being, replicate Hwang and Won's (2021) results. The results of the present study, however, add that social cues can be used to enhance the effect of chatbot identity on the generation of diverse ideas, and possibly on the number of ideas. The use of social cues may have particularly emphasized the identity of the chatbot brainstorming partner during the brainstorming session, which may have enhanced certain advantages of brainstorming with a chatbot over brainstorming with a human, especially if an upward social comparison took place (Michinov et al., 2015).

There are also several limitations to this study. Firstly, the additional measures (perceived humanness, homophily, and social presence) did not differ significantly between the brainstorming partner and social cues conditions. Thus, the differences between the four conditions in the study may have been too minimal, posing a threat to the internal validity of the study. Despite these minimal differences, the identity of the brainstorming partner and the use of social cues influenced the outcome of collaborative brainstorming sessions. It is therefore also possible that these were not appropriate constructs to measure the differences between brainstorming partners and use of social cues. Another limitation of the current study is the lack of control over the execution of the brainstorming task, because it was automated and administered entirely online. Participants' home environment may have been distracting during the brainstorming sessions, posing a threat to the internal validity of the study. Automating the brainstorming task could also negatively impact the credibility of the human brainstorming partner. Moreover, the sample of this study, consisting merely of students, threatens the generalizability of the results. Lastly, using an automated script that does not consider the user's input could pose a threat to the study's external validity, because it remains to be seen whether an actual AI could be developed to effectively function as a brainstorming partner in practice.

Future work is needed to uncover the mechanisms other than evaluation apprehension by which chatbots can enhance brainstorming performance. A higher level of motivation to brainstorm with a chatbot because of its novelty could be a first possible factor (Paulus et al., 2002; Hwang and Won, 2021). Increased motivation has shown a positive influence on the outcome of brainstorming sessions (Paulus et al., 2002). A second possible explanation is compensatory behavior. It is known that interactions with chatbots work according to fixed rules and data (Bittner and Shoury, 2019), and they are therefore perceived to be less skilled than human beings. Possibly, participants performed better because they compensated for this perceived lack of skill of a chatbot. The participants in the chatbot condition knew that the ideas the chatbot shared came from an existing dataset, meaning that they were the only ones who generated new ideas and contributed to the brainstorming session, while in the human condition they thought they were brainstorming in real-time with another person. However, one might then also expect a social facilitation effect when participants thought they were collaborating with someone in real-time, which should have led to higher performance in the human condition instead (cf. Paulus et al., 2013). A third explanation is the opposite of this compensation behavior, namely that the chatbot was seen as a more skillful partner than a human, resulting in an upward social comparison (cf. Michinov et al., 2015), although this has thus far only been observed with different human brainstorming partners, and not when a computer system generated ideas automatically (Dugosh and Paulus, 2005). It may be that the design of our interface created a sense of social presence that has facilitated social comparison. Fourth and finally, the potential role of behavioral disinhibition might also merit further investigation (Eysenck, 1995). Possibly, chatbots do not necessarily reduce a fear of being evaluated negatively, but rather elicit a feeling that one can simply say anything to a chatbot (cf. Hwang and Won, 2021). Understanding better what factors drive a chatbot's effects on brainstorming can help optimize their design as brainstorming partners.

Herewith, the contribution of the present study is that collaboratively brainstorming with a chatbot, compared to with an alleged human being, causes the generation of more ideas with more diversity. This effect can be reinforced by providing social cues, but is not explained by the conjectured reduction in evaluation apprehension.

## Data Availability Statement

The original contributions presented in the study are included in the article/Supplementary material, further inquiries can be directed to the corresponding author.

## Ethics Statement

Ethical review and approval was not required for the study on human participants in accordance with the local legislation and institutional requirements. The patients/participants provided their written informed consent to participate in this study.

## Author Contributions

BW, JW, and AR developed the theory and method and wrote the manuscript. BW and JW developed the materials. BW collected and processed the data and conducted the analysis. All authors contributed to the article and approved the submitted version.

## Conflict of Interest

The authors declare that the research was conducted in the absence of any commercial or financial relationships that could be construed as a potential conflict of interest.

## Publisher's Note

All claims expressed in this article are solely those of the authors and do not necessarily represent those of their affiliated organizations, or those of the publisher, the editors and the reviewers. Any product that may be evaluated in this article, or claim that may be made by its manufacturer, is not guaranteed or endorsed by the publisher.
