# Supplementary material for: Electronic Brainstorming With a Chatbot Partner: A Good Idea Due to Increased Productivity and Idea Diversity
Source: Front Artif Intell. 2022 Sep 23;5:880673. doi: 10.3389/frai.2022.880673 (PMC9537607; doi:10.3389/frai.2022.880673)
Supplement: Supplementary file 1 [file Table_1.DOCX]

| Interval | Idea |
| --- | --- |
| 00.24 | Providing information about birth control in third world countries |
| 01.00 | Making birth control free |
| 01.48 | Better education for women in third world countries |
| 02.36 | No more child benefit from 2 children onwards |
| 03.00 | Legalize abortion and euthanasia everywhere |
| 04.12 | Distribute the population evenly over the earth |
| 04.36 | Start building food stocks already |
| 05.15 | Appoint David Attenborough as Global Minister for Overpopulation |
| 05.48 | Refusing international students in the Netherlands |
| 06.00 | What are those kinds of ideas… |
| 06.10 | Sorry, that was wrong |
| 06.24 | Build new land in the sea, such as Flevoland |
| 07.14 | Building tree houses |
| 08.18 | Making adoption more attractive: free food for 5 years |
| 08.40 | Living in spaceships |
| 09.30 | Artificially cultivating new raw materials |
