# Supplementary material for: Electronic Brainstorming With a Chatbot Partner: A Good Idea Due to Increased Productivity and Idea Diversity
Source: Front Artif Intell. 2022 Sep 23;5:880673. doi: 10.3389/frai.2022.880673 (PMC9537607; doi:10.3389/frai.2022.880673)
Supplement: Supplementary file 2 [file Data_Sheet_1.PDF]

Bachelorscriptie Britt Wieland

Start of Block: Introductie van het onderzoek

Q12 Bedankt voor je deelname aan dit onderzoek. Middels dit onderzoek wordt er wetenschappelijk inzicht verkregen in collaboratieve brainstormsessies. Dit onderzoek wordt uitgevoerd voor mijn bachelorscriptie aan Tilburg University.

Je deelname aan dit onderzoek is geheel vrijwillig. Je kunt je ten alle tijde, ongeacht je reden, het onderzoek verlaten door de vragenlijst weg te klikken in de webbrowser. Wanneer je deelneemt aan het onderzoek via de proefpersonenpool van Tilburg University, zal je na afronding van het onderzoek automatisch 0.5 punt ontvangen. De antwoorden die je geeft, worden volledig anoniem verwerkt. De verkregen data zullen conform het beleid van Tilburg University 10 jaar worden bewaard. Er zijn geen risico's verbonden aan een deelname aan dit onderzoek.

Je deelname aan dit onderzoek zal ongeveer 15 minuten duren. Je zal eerst wat vragen krijgen over jouw creativiteit. Daarna zal je samen met een brainstormpartner een korte brainstormtaak uitvoeren. Hierna krijg je een aantal vragen over het verloop van de brainstormsessie. Voor vragen over het onderzoek kun je mailen naar b.b.wieland@tilburguniversity.edu.

End of Block: Introductie van het onderzoek

Start of Block: Voorwaarden en consent

Q17 Lees de onderstaande informatie zorgvuldig door en geef onderaan de lijst aan of je akkoord gaat met de voorwaarden.

Titel: Collaboratieve brainstormsessies
Naam van de onderzoeker: Britt Wieland
Doel: Het bestuderen van de werking van collaboratieve brainstormsessies.

Ik wil je vragen om akkoord te gaan met onderstaande verklaringen:

- Ik ben 16 jaar of ouder.
- Ik heb de informatie aan het begin van het onderzoek gelezen waarin het onderzoeksproject wordt uitgelegd (informatie over de deelname, procedure en risico's).
- Ik heb de mogelijkheid gekregen om vragen te stellen en al deze vragen zijn naar mijn tevredenheid beantwoord.
- Ik begrijp dat ik mij zonder het geven van een reden ten alle tijden kan terugtrekken.
- Ik ga er mee akkoord dat de onderzoeksgegevens voor een periode van 10 jaar worden bewaard en dat de gegevens van mij worden verwerkt zonder dat deze verbonden worden aan mijn persoonlijke gegevens.
- Ik begrijp dat de gegevens en data gebruikt worden voor het schrijven van een bachelorscriptie.
- Ik begrijp dat ik kan mailen naar b.b.wieland@tilburguniversity.edu wanneer ik vragen heb over het doel of de procedure van dit onderzoek.
- Ik ga vrijwillig akkoord om deel te nemen aan dit onderzoek.

Q16 oGa je akkoord met een deelname aan dit onderzoek?

○ Ja, ik ga akkoord en zal deelnemen aan het onderzoek. (1)

○ Nee, ik ga niet akkoord en zal niet deelnemen aan het onderzoek. (2)

End of Block: Voorwaarden en consent

Start of Block: Demografische gegevens

Q20 Wat is je leeftijd?

____

Q18 Wat is je geslacht?

- Man (1)
- Vrouw (2)
- Anders (3) ____

Q43 Ben je student aan de Universiteit van Tilburg?

- Ja (1)
- Nee (2)

End of Block: Demografische gegevens

Start of Block: Short Scale of Creative Self (Karwowski,2016)

Q2 De volgende uitspraken gaan over creativiteit. Geef aan in hoeverre je het eens/oneens bent met de uitspraken.

	helemaal mee oneens (1)	mee oneens (2)	neutraal (3)	mee eens (4)	helemaal mee eens (5)
Ik denk dat ik een creatief persoon ben. (1)	○	○	○	○	○
Mijn creativiteit is belangrijk voor wie ik ben. (2)	○	○	○	○	○
Ik weet dat ik zelf gecompliceerde problemen efficiënt kan oplossen. (3)	○	○	○	○	○
Ik vertrouw op mijn creatieve vaardigheden (4)	○	○	○	○	○
Mijn verbeeldingsvermogen en vindingrijkheid onderscheiden mij van mijn vrienden. (5)	○	○	○	○	○
Ik heb vaak bewezen dat ik goed om kan gaan met moeilijke situaties. (6)	○	○	○	○	○
Een creatief persoon zijn is belangrijk voor me. (7)	○	○	○	○	○
Ik ben er zeker van dat ik kan omgaan met problemen waar creatief denkvermogen voor nodig is. (8)	○	○	○	○	○
Ik ben goed in het bedenken van originele probleemoplossingen. (9)	○	○	○	○	○
Creativiteit is een belangrijk onderdeel van mezelf. (10)	○	○	○	○	○

Vindingrijkheid is een belangrijke eigenschap van mij. (11)	○	○	○	○	○

End of Block: Short Scale of Creative Self (Karwowski,2016)

Start of Block: Probleemstelling

Q13 **Probleemstelling** Door een steeds verder toenemende bevolkingsgroei raakt de aarde overvol en overbelast. Dit leidt tot onder andere milieuvervuiling, een verlies aan biodiversiteit en gezondheidsproblemen zoals de huidige pandemie. Een steeds verder toenemende bevolkingsgroei kan leiden tot overbevolking. Er is sprake van overbevolking wanneer de balans tussen de bevolking en de bestaansmiddelen die beschikbaar zijn op de aarde verstoord is. Overbevolking wordt beschreven als een van de grootste bedreigingen voor het voortbestaan van de mensheid. Ook in Nederland dreigt er overbevolking; Nederland is na Taiwan en Bangladesh het dichtstbevolkte land van de wereld. Dit probleem is vandaag de dag goed merkbaar. Denk maar aan de huidige woningcrisis, de stikstofcrisis en de groeiende tekorten in de zorg.

End of Block: Probleemstelling

Start of Block: Brainstormtaak & regels - mens conditie

Lees de onderstaande informatie zorgvuldig door.

Brainstormtaak
Je gaat samen met een student assistent van Tilburg University 10 minuten lang via een chatvenster brainstormen over de probleemstelling: *Hoe kunnen we in Nederland en de rest van de wereld omgaan met de dreigende overbevolking?* Je kunt je ideeën delen door in het chatvenster op het verstuur-icoon te klikken. Verstuur telkens 1 idee per bericht. Let op: verstuur alleen je ideeën, geen andere berichten. Jij en de andere student zullen elkaars ideeën ontvangen. Je hoeft niet te wachten op een idee van de andere student voordat je een nieuw idee verstuurt. Na 10 minuten sluit het chatvenster zich vanzelf af.

Brainstormregels
Bij brainstormen is het de bedoeling om zo veel mogelijk verschillende ideeën te verzinnen. De kwaliteit van de ideeën is hierbij niet van belang en ideeën mogen niet beoordeeld worden

tijdens de brainstormsessie.

Page Break

End of Block: Brainstormtaak & regels - mens conditie

Start of Block: Wachten op student

Q41 Wachten op de student assistent...

Q42 Timing
First Click (1)
Last Click (2)
Page Submit (3)
Click Count (4)

End of Block: Wachten op student

Start of Block: Chatvenster mens man + sociale cues

JS

Q14 **Brainstorm-onderwerp:** Hoe kunnen we in Nederland en de rest van de wereld omgaan met de dreigende overbevolking?

Let op: verstuur alleen ideeën naar elkaar.

Q28 Timing
First Click (1)
Last Click (2)
Page Submit (3)
Click Count (4)

End of Block: Chatvenster mens man + sociale cues

Start of Block: Chatvenster mens vrouw + sociale cues

JS

Q29 **Brainstorm-onderwerp:** Hoe kunnen we in Nederland en de rest van de wereld omgaan met de dreigende overbevolking?

Let op: verstuur alleen ideeën naar elkaar.

Q30 Timing
First Click (1)
Last Click (2)
Page Submit (3)
Click Count (4)

End of Block: Chatvenster mens vrouw + sociale cues

Start of Block: Chatvenster mens man - zonder sociale cues

JS

Q32
Brainstorm-onderwerp: Hoe kunnen we in Nederland en de rest van de wereld omgaan met de dreigende overbevolking?

Let op: verstuur alleen ideeën naar elkaar.

Q33 Timing
First Click (1)
Last Click (2)
Page Submit (3)
Click Count (4)

End of Block: Chatvenster mens man - zonder sociale cues

Start of Block: Chatvenster mens vrouw - zonder sociale cues

JS

Q34
Brainstorm-onderwerp: Hoe kunnen we in Nederland en de rest van de wereld omgaan met de dreigende overbevolking?

Let op: verstuur alleen ideeën naar elkaar.

Q35 Timing
First Click (1)
Last Click (2)
Page Submit (3)
Click Count (4)

End of Block: Chatvenster mens vrouw - zonder sociale cues

Start of Block: Einde brainstormsessie

Q40 De brainstormsessie is voorbij. Je zal nu wat vragen krijgen over het verloop van de brainstormsessie.

End of Block: Einde brainstormsessie

Start of Block: Schaal voor evaluatievrees (Bolin & Neuman, 2006)

Q3 De volgende uitspraken gaan over de brainstormsessie. Geef aan in hoeverre je het eens/oneens bent met de volgende uitspraken.

	helemaal mee oneens (1)	mee oneens (2)	neutraal (3)	mee eens (4)	helemaal mee eens (5)
Mijn brainstormpartner en ik keken naar elkaars verstuurde ideeën. (1)	○	○	○	○	○
Ik voelde me op mijn gemak tijdens de brainstormsessie. (2)	○	○	○	○	○
Mijn brainstormpartner was erg kritisch in zijn/haar reactie op mijn ideeën. (3)	○	○	○	○	○
Ik was bang dat mijn brainstormpartner slecht over mij zou gaan denken. (4)	○	○	○	○	○
Ik zou niet willen dat mijn naam wordt gehecht aan mijn ideeën. (5)	○	○	○	○	○
Ik bleef maar denken dat mijn brainstormpartner mijn ideeën zou bekritiseren. (6)	○	○	○	○	○
Ik heb niet al mijn ideeën verstuurd, omdat ik niet wilde dat mijn brainstormpartner dacht dat ik raar	○	○	○	○	○

of gek was. (7)	

End of Block: Schaal voor evaluatievrees (Bolin & Neuman, 2006)

Start of Block: Schaal voor social presence (Go & Sundar, 2019)

Q6 De volgende uitspraken gaan over je brainstormpartner en de brainstormsessie. Geef aan in hoeverre je het eens/oneens bent met de uitspraken.

	helemaal mee oneens (1)	mee oneens (2)	neutraal (3)	mee eens (4)	helemaal mee eens (5)
Er was een gevoel van menselijk contact met mijn brainstormpartner. (1)	○	○	○	○	○
Er was een gevoel van persoonlijke communicatie met mijn brainstormpartner. (2)	○	○	○	○	○
Er heerste een gevoel van gezelligheid bij de brainstormsessie. (3)	○	○	○	○	○
Er heerste een gevoel van warmte bij de brainstormsessie. (4)	○	○	○	○	○
Er was een gevoel van gevoeligheid bij de brainstormsessie. (5)	○	○	○	○	○

End of Block: Schaal voor social presence (Go & Sundar, 2019)

Start of Block: Schaal voor waargenomen homophily (Go & Sundar, 2019)

Q11 De volgende uitspraken gaan over je brainstormpartner. Geef aan in hoeverre je het eens/oneens bent met de uitspraken.

	helemaal mee oneens (1)	mee oneens (2)	neutraal (3)	mee eens (4)	helemaal mee eens (5)
Mijn brainstormpartner is vergelijkbaar met mezelf. (1)	○	○	○	○	○
Mjin brainstormpartner denkt hetzelfde als ik. (2)	○	○	○	○	○
Mijn brainstormpartner lijkt op mij. (3)	○	○	○	○	○
Mijn brainstormpartner gedraagt zich zoals ik. (4)	○	○	○	○	○

End of Block: Schaal voor waargenomen homophily (Go & Sundar, 2019)

Start of Block: Schaal voor waargenomen menselijkheid (Hendriks et al., 2020)

Q11 De volgende stellingen gaan over je brainstormpartner. Geef aan in hoeverre je het eens bent met de volgende stellingen. *Ik vond mijn brainstormpartner*:

	1 (1)	2 (2)	3 (3)	4 (4)	5 (5)	6 (6)	7 (7)	
extreem onmenselijk	○	○	○	○	○	○	○	extreem menselijk
extreem onbekwaam	○	○	○	○	○	○	○	extreem bekwaam
extreem onbedachtzaam	○	○	○	○	○	○	○	extreem bedachtzaam
extreem onbeleefd	○	○	○	○	○	○	○	extreem beleefd
extreem niet-responsief	○	○	○	○	○	○	○	extreem responsief
extreem niet-betrokken	○	○	○	○	○	○	○	extreem betrokken

End of Block: Schaal voor waargenomen menselijkheid (Hendriks et al., 2020)

Start of Block: Debriefing mens

Bedankt voor je deelname aan dit onderzoek! Tijdens de brainstormsessie heb je gechat met een automatische chatbot die ideeën uit een dataset met jou deelde. Er was dus geen andere student aanwezig. De gepresenteerde identiteit van de chatbot werd tijdens het onderzoek verborgen gehouden. In deze studie wordt namelijk onderzocht in hoeverre de perceptie van de identiteit van een brainstormpartner (mens of robot) de uitkomst van een brainstormsessie beïnvloedt. Praat alsjeblieft niet met anderen (toekomstige participanten) over het doel van de studie.

Als je meer wilt weten over het onderzoek of de uitkomst van het onderzoek, kan je mailen naar b.b.wieland@tilburguniversity.edu

Page Break

End of Block: Debriefing mens

Start of Block: Brainstormtaak & regels - robot conditie

Lees de onderstaande informatie zorgvuldig door.

Brainstormtaak
Je gaat samen met kunstmatige intelligentie in de vorm van een chatbot 10 minuten lang via een chatvenster brainstormen over de probleemstelling: *Hoe kunnen we in Nederland en de rest van de wereld omgaan met de dreigende overbevolking?* Een chatbot is een technologie die gebaseerd is op voorgeschreven regels en gebruik maakt van natuurlijke taal. De chatbot zal ideeën met jou delen die gebaseerd zijn op eerder verzamelde data. Jouw ideeën zijn zichtbaar voor de chatbot. Je kunt je ideeën delen door in het chatvenster op het verstuur-icoon te klikken. Verstuur telkens 1 idee per bericht. Let op: verstuur alleen je ideeën, geen andere berichten. Je hoeft niet te wachten op een idee van de chatbot voordat je een nieuw idee verstuurt. Na 10 minuten sluit het chatvenster zich vanzelf af.

Brainstormregels
Bij brainstormen is het de bedoeling om zo veel mogelijk verschillende ideeën te verzinnen. De kwaliteit van de ideeën is hierbij niet van belang en ideeën mogen niet beoordeeld worden tijdens de brainstormsessie.

End of Block: Brainstormtaak & regels - robot conditie

Start of Block: Chatvenster robot - zonder sociale cues

JS

Q36
Brainstorm-onderwerp: Hoe kunnen we in Nederland en de rest van de wereld omgaan met de dreigende overbevolking?

Let op: verstuur alleen ideeën naar elkaar.

Q37 Timing
First Click (1)
Last Click (2)
Page Submit (3)
Click Count (4)

End of Block: Chatvenster robot - zonder sociale cues

Start of Block: Chatvenster robot + met sociale cues

JS

Q26 **Brainstorm-onderwerp:** Hoe kunnen we in Nederland en de rest van de wereld omgaan met de dreigende overbevolking?

Let op: verstuur alleen ideeën naar elkaar.

Q38 Timing
First Click (1)
Last Click (2)
Page Submit (3)
Click Count (4)

End of Block: Chatvenster robot + met sociale cues

Start of Block: Debriefing robot

Q39 Bedankt voor je deelname aan dit onderzoek! Tijdens de brainstormsessie heb je gechat met een automatische chatbot die ideeën uit een dataset met jou deelde. In deze studie wordt onderzocht in hoeverre de perceptie van de identiteit van een brainstormpartner (mens of robot) de uitkomst van een brainstormsessie beïnvloedt. Praat alsjeblieft niet met anderen (toekomstige participanten) over het doel van de studie.

Als je meer wilt weten over het onderzoek of de uitkomst van het onderzoek, kan je mailen naar b.b.wieland@tilburguniversity.edu

End of Block: Debriefing robot
